# Standards for Deriving Nonhuman Primate-Induced Pluripotent Stem Cells, Neural Stem Cells and Dopaminergic Lineage

**DOI:** 10.3390/ijms19092788

**Published:** 2018-09-17

**Authors:** Guang Yang, Hyenjong Hong, April Torres, Kristen E. Malloy, Gourav R. Choudhury, Jeffrey Kim, Marcel M. Daadi

**Affiliations:** 1 Southwest National Primate Research Center, Texas Biomedical Research Institute, San Antonio, TX 78245-0549, USA; gyang@txbiomed.org (G.Y.); HHong@txbiomed.org (H.H.); aatorres@txbiomed.org (A.T.); KMalloy@txbiomed.org (K.E.M.); GChoudhury@txbiomed.org (G.R.C.); JKim@txbiomed.org (J.K.); 2 Department of Radiology, Long School of Medicine, University of Texas Health San Antonio, San Antonio, TX 78229-3900, USA

**Keywords:** nonhuman primate iPSC, iPSC repository, iPSC characterization, standards for pluripotency, differentiation of dopaminergic neurons, Parkinson’s disease

## Abstract

Humans and nonhuman primates (NHP) are similar in behavior and in physiology, specifically the structure, function, and complexity of the immune system. Thus, NHP models are desirable for pathophysiology and pharmacology/toxicology studies. Furthermore, NHP-derived induced pluripotent stem cells (iPSCs) may enable transformative developmental, translational, or evolutionary studies in a field of inquiry currently hampered by the limited availability of research specimens. NHP-iPSCs may address specific questions that can be studied back and forth between in vitro cellular assays and in vivo experimentations, an investigational process that in most cases cannot be performed on humans because of safety and ethical issues. The use of NHP model systems and cell specific in vitro models is evolving with iPSC-based three-dimensional (3D) cell culture systems and organoids, which may offer reliable in vitro models and reduce the number of animals used in experimental research. IPSCs have the potential to give rise to defined cell types of any organ of the body. However, standards for deriving defined and validated NHP iPSCs are missing. Standards for deriving high-quality iPSC cell lines promote rigorous and replicable scientific research and likewise, validated cell lines reduce variability and discrepancies in results between laboratories. We have derived and validated NHP iPSC lines by confirming their pluripotency and propensity to differentiate into all three germ layers (ectoderm, mesoderm, and endoderm) according to standards and measurable limits for a set of marker genes. The iPSC lines were characterized for their potential to generate neural stem cells and to differentiate into dopaminergic neurons. These iPSC lines are available to the scientific community. NHP-iPSCs fulfill a unique niche in comparative genomics to understand gene regulatory principles underlying emergence of human traits, in infectious disease pathogenesis, in vaccine development, and in immunological barriers in regenerative medicine.

## 1. Introduction

Regenerative medicine focuses on creating vital functional cells to repair or replace tissue or organs damaged by disease, injury, or congenital defects. This approach may provide treatments for currently intractable diseases. The burgeoning of this medical approach has been galvanized by the potential of induced pluripotent stem cells (iPSCs) [1,2,3]. The possibility to generate specialized organ-specific differentiated cells using human somatic cells induced to become iPSCs opens the door for individualized cell therapy. Personalized or autologous cell therapy would be ideal for treating various diseases or injuries, such as heart attacks, diabetes, stroke, neurodegenerative diseases, and others, as it would obviate the need for immunosupressants and may provide better functional integration of autologous cells in the diseased or injured tissues. Standardized and well-defined stable iPSC lines and animal model resources may play a critical role to support efforts in the scientific community and industry to move forward this therapeutic strategy into clinical use.

Recent progress in stem cell biology revealed that tissue specific mature adult cells retain the capacity to be reprogrammed into pluripotent stem cells [1,2,3]. A set of transcription factors including *OCT3/4*, *SOX2*, *KLF4*, *L-MYC*, *and LIN28*, once introduced into somatic cells, such as skin fibroblasts, is capable of reprogramming them back to a pluripotent stage or to iPSCs that have characteristics similar to human embryonic stem cells. These cells have virtually an unlimited capacity to proliferate and differentiate into all cell types of the body and thus bring new dimensions to regenerative biology.

Following the generation of human iPSC, [1,2] the derivation of the NHP-iPSCs has been reported from various NHP species, including the rhesus macaque, the marmoset, and the baboon [4,5,6,7,8,9,10,11,12]. IPSCs from NHP play a unique role in cell biology and regenerative medicine as they offer the missing link between rodent and human studies aimed at understanding the basic mechanisms controlling pluripotency, cell fate determination, and pathogenesis of diseases. NHP-iPSCs can serve as a source of differentiated derivatives that can be used to address important questions in developmental, evolutionary, and comparative biology of primates [13,14,15,16]. When used as a source of cells for regenerative medicine, NHP-iPSCs serve an invaluable role in translational autologous and allogeneic experimental cell therapy. Given the similarities between humans and NHPs in the complexity of the immune system, the use of iPSCs is most relevant to understanding how the grafted cells functionally integrate and their subtle interactions with the immune system. In this study, we report the establishment of standards for deriving iPSCs from NHP and their differentiation into neural lineages using the marmoset as a model.

## 2. Results

### 2.1. Derivation of Marmoset iPSC Using Human Reprogramming Factors

**Figure 1:**
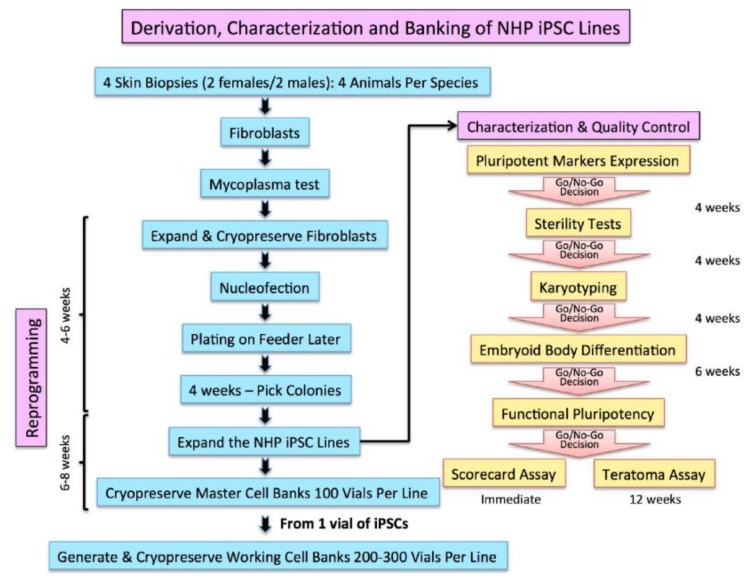
Process of generation, characterization, and validation of iPSC lines. Go/no-go workflow to generate, characterize, and bank nonhuman primate (NHP)-induced pluripotent stem cells (iPSC) lines.

The generation of iPSCs from NHP skin biopsies from marmosets was performed according to the quality control program outlined in Figure 1. Given the homology in sequence between marmosets and humans (>95%), we used human reprogramming factors and asked whether they would be efficient in reprogramming marmoset fibroblasts. We used a non-integrative episomal vector system expressing the reprogramming factors *OCT3/4*, shRNA for *p53*, *SOX2*, *KLF4*, *L-MYC*, and *LIN28*, which offers zero-footprint [17]. We selected 12 reprogrammed colonies and expanded for three passages before banking them; three clones were further characterized for their pluripotency. From these clones, we selected the optimal cell lines that met the criteria outlined in Figure 1. If a clone failed the Go-No-Go quality control criteria, a new clone was thawed and expanded. We consistently obtained qualified iPSCs lines among the selected clones.

### 2.2. Characterization and Banking of the NHP iPSCs

**Figure 2:**
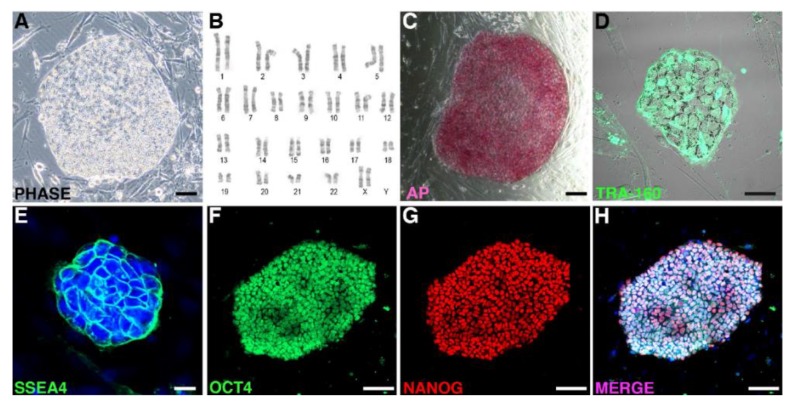
Characterization of the NHP-iPSC lines. (**A**) Example of the marmoset iPSC colony derived by reprograming skin fibroblast using the episomal approach showing a normal karyotype at passage 16 (**B**). The iPSC colonies express pluripotent markers, including alkaline phosphatase (**C**), TRA-160 (**D**), SSEA4 (**E**), OCT-4 (**F**), and NANOG (**G**). Scale bars: 100 μm in (**A**,**C**,**F**–**H**); 50 μm in (**D**); and 20 μm in (**E**).

We first assessed the colonies based on their morphologies. Colonies with human ES cell-like morphology were selected and expanded. All iPSC lines were cultured for several passages. The iPSC colonies displayed typical ES cell-like morphology (Figure 2A). The authentication of these iPSCs is key for ensuring reliable scientific research. We performed cytogenetic analysis to continuously monitor genomic stability in each iPSC line using karyotype analysis (Figure 2B) as a baseline screen. We selected a total of 20 cells per culture every 5 to 10 passages per cell line to screen for microscopic aberrations of over 5 Mb chromosomes, including aneuploidy, inversions, duplications, deletions, and translocations. None of the clones showed abnormalities and we selected two clones: CJ01 and CJ02. The selected iPSC lines were verified for the expression of the pluripotency markers including alkaline phosphatase expression assay (Figure 2C) and TRA-1-60, SSEA4, OCT4, and NANOG by immunocytochemistry (Figure 2D–H).

**Figure 3:**
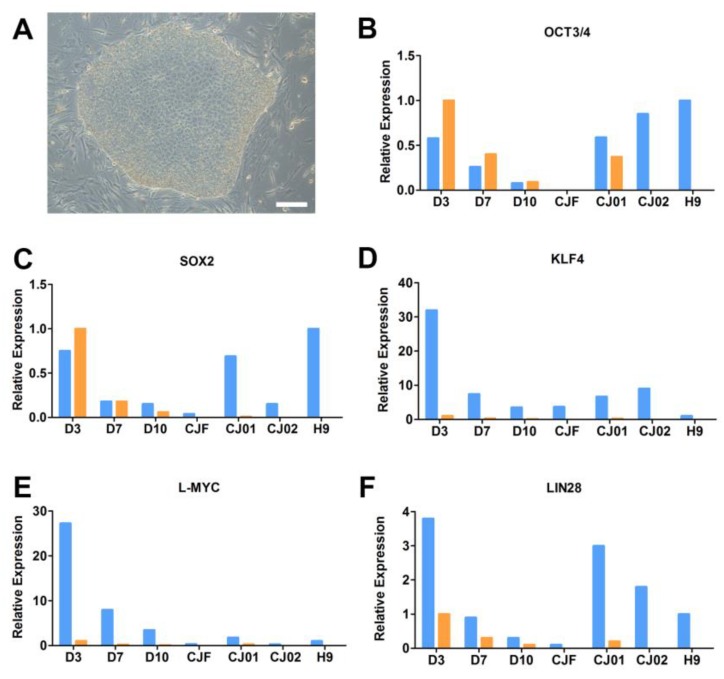
Generation of the iPSC line CJ01 and CJ02 from marmoset skin biopsies. (**A**) Phase contrast image of generated marmoset iPSC, CJ02 iPSC passage 2; (**B**) quantitative RT-PCR results of *OCT3/4*, (**C**) *SOX2*, (**D**) *KLF4*, (**E**) *L-MYC*, and (**F**) *LIN28*. Blue bar indicates the expression of genes by coding region-specific primers and orange bar indicate expression of pluripotent genes by plasmid-specific primers. D3, D7, and D10 indicate RNA collected at days 3, 7, and 10, respectively, after transfection of reprogramming factors. Abbreviations: CJF; marmoset skin fibroblast, CJ01 and CJ02 are marmoset iPSC clones generated, H9: human embryonic stem cell line H9. Scale bar in A: 200 μm

To further characterize and validate the CJ01 and CJ02 clones, we performed Q-PCR to quantify the endogenous coding pluripotent genes *OCT3/4*, *SOX2*, *KLF4*, *L-MYC*, and *LIN28* versus their exogenous vector-mediated expression (Figure 3). The data showed the CJ01 clone had some exogenous expression of *OCT3/4* and minimal expression of *LIN28*, while CJ02 was devoid of any vector-based expression (Figure 3). This is a further step in the Go-No-Go process to eliminate any lines with integration of the reprograming factors.

### 2.3. The Q-PCR Scorecard Standard to Select Pluripotent iPSC Lines

**Figure 4:**
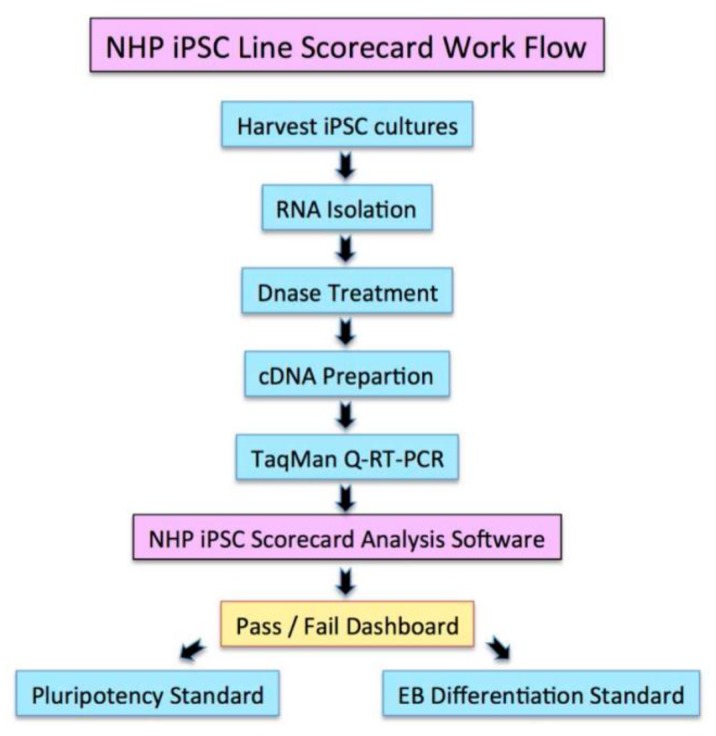
Workflow of the Scorecard: representation of the steps leading to the pluripotency and differentiation standard tests. Left, the pluripotency; and right, the embryoid body (EB) differentiation standard test.

**Figure 5:**
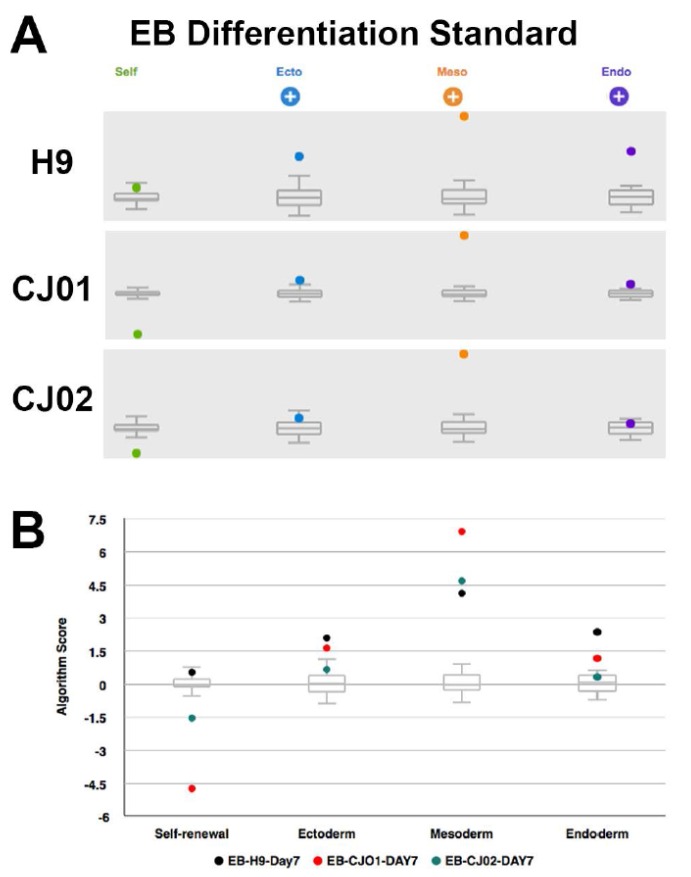
Score box plot from scorecard analysis for differentiation potential. (**A**) Scorecard results of H9, CJ01, and CJ02 iPSC differentiation tendency to the undifferentiated reference set. The samples were seven-day old embryoid bodies (EBs). The EBs downregulated pluripotent genes (green) as shown with the negative (−) sign for CJ01 and CJ02 and significantly upregulated EC, ME, and ED as shown with the (+) sign. (**B**) Score box plot represents comparison of all samples. Black, red, and green dots indicate seven-day old EBs from H9, CJ01, and CJ02, respectively. The range of scores for the undifferentiated reference set is indicated by the gray box score on the 0 score line.

**Figure 6:**
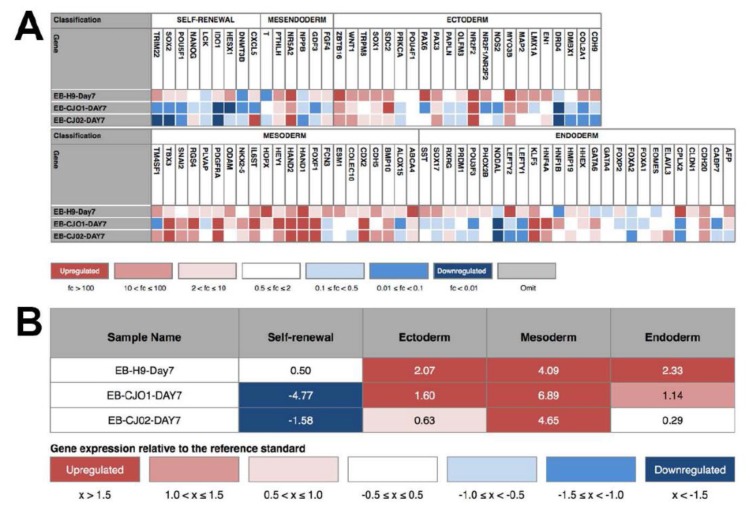
Heat map and score table from scorecard analysis for differentiation potential. (**A**) Heat map of expression of self-renewal and three germ layer markers by the seven-day old EBs differentiated from the H9, CJ01, and CJ02; (**B**) Score table showing scores derived from statistical comparison of the sample expression profile to that of the undifferentiated reference set. Colors correlate to the fold change in expression of the indicated gene relative to the undifferentiated reference set.

Teratoma formation has been used as a pluripotency assay; however, results from this assay suffer from variability and since they are not quantitative, making decisions on the quality of cell lines is difficult [18,19,20]. Validated genomic approaches [21,22] have been used as a quantitative and quick way to assess the quality and for functional pluripotency. We used a genomic assay, the Scorecard, to quantify according to a flowchart (Figure 4) the quality of the iPSCs through the evaluation of the pluripotency molecular signature and the functional pluripotency signatures [22,23]. We tested the Scorecard on our iPSC lines to assess pluripotency and propensity to differentiate into the three germ layers: ectoderm (EC), mesoderm (ME), and endoderm (ED). The marmoset iPSC clones (CJ01, CJ02) were differentiated into embryoid bodies (EBs) for seven days in vitro (DIV) passed the test, as indicated in Figure 5 by the sign (+) with significant downregulation of pluripotency genes and significant upregulation of the EC, ME, and ED genes (Figure 6). Quantitative gene expression signature assay demonstrated the identity and expression profile of the lineage-specific genes (Figure 6)

### 2.4. Derivation of Neural Stem Cells (NSCs) from iPSCs and Their Differentiation into Dopaminergic Lineage

**Figure 7:**
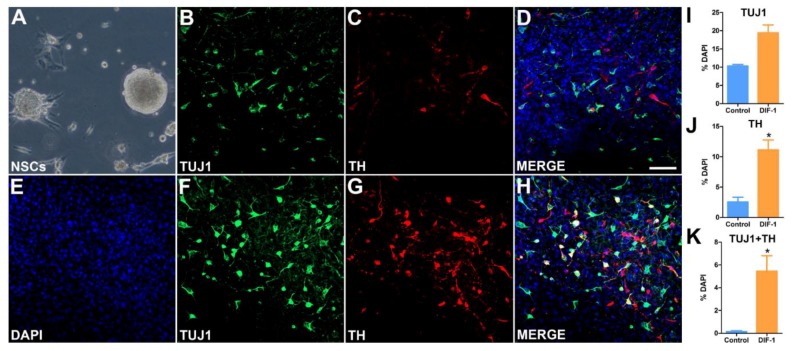
Differentiation of marmoset iPSCs into dopaminergic neurons. (**A**) Phase contrast microphotograph of NSCs derived from marmoset iPSCs. (**B**–**D**) NSCs differentiated for seven days in control media showing the neuronal marker β-III Tubulin (TuJ1, green, (**B**)), tyrosine hydroxylase (TH^+^ in red (**C**), the rate limiting enzyme of dopamine biosynthesis), and merged image of all channels (**D**). (**E**–**H**) Representative immunofluorescence photos of NSCs differentiated for seven days under dopamine-inducing conditions in Dopamine Inducing Factor 1 (DIF1) media showing an increased number of TH^+^ neurons (red (**G**)). (**I**) Quantitative analysis of TUJ1^+^ neurons in control and in DIF1 media showing an increase of percentage of neurons under DIF1 conditions. (**J**) The number of TH^+^ significantly increased in DIF1 media compared with control. (**K**) Quantitative analysis of TUJ1^+^/TH^+^ double-labeled neurons in control and DIF1 media. The number of TH^+^ neurons was significantly high under the dopamine inducing conditions in DIF1 media compared to control. * *p* < 0.05 vs. control. Scale bar 50 μm.

**Figure 8:**
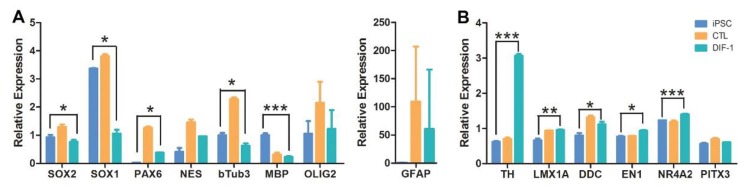
TaqMan quantitative RT-PCR analysis of dopamine-differentiated neural stem cells (NSCs). (**A**) Expression level of genes markers of NSCs and their progeny (*NESTIN*, *SOX1*, *SOX2 PAX6*, *β-Tub-III*, *MBP*, *Olig-2*, *GFAP*) under control media (orange) and DIF1 media derived dopaminergic differentiation (green) and from pluripotent undifferentiated CJ01 iPSC (blue); (**B**) expression level of genes markers of the midbrain dopaminergic lineage (*TH*, *LMX1A*, *EN1*, *NURR1*, *PITX3*, and dopamine decarboxylase (*DDC*) enzyme that convert L-DOPA to dopamine) in NSC differentiated under control media (orange) or DIF1 media (green) and from undifferentiated CJ01 iPSC (blue), confirming the increase in the dopaminergic neuronal differentiation. * *p* < 0.05, ** *p* < 0.01, *** *p* < 0.001; *n* = 3. Error bars represent relative expression ± S.D.

The efficiency of pluripotent iPSC lines to differentiate into specific cell types varies between cell lines. Both CJ01 and CJ02 were cultured in the NSC medium (NN1) to derive the self-renewable NSCs. The NN1 medium is supplemented by the growth factors EGF and bFGF, which stimulates neural precursors to proliferate and generate progenies assembled in a spherical shape. The spheres of NSCs were passaged weekly to generate a large number of NSCs, confirming their self-renewable ability. The NSCs were readily differentiated to neurons confirming their competency to generate neural lineages. To determine their competency to differentiate into dopaminergic lineage, we treated the NSCs with the dopaminergic differentiation factors DIF1. There was a significant increase in the number of TH-positive neurons under DIF1 conditions as demonstrated by immunocytochemical analysis (Figure 7). To further confirm the neural and dopaminergic lineage differentiation, we performed Q-PCR on key genes that are characteristic of NSC progeny and the midbrain dopaminergic lineage. The data demonstrated the neural and dopaminergic identity of the cell line, and the increase in TH expression under the dopamine inducing condition DIF1 (Figure 8).

## 3. Discussions

We report the derivation of qualified pluripotent marmoset iPSCs, using the non-integrative episomal approach. We describe for the first time a genomic approach to set standards for validating the pluripotency in NHP-iPSC lines. This is based on a set of genes that are definitive markers of pluripotency and propensity to generate the three germ layers. We also describe the generation of NSCs and their differentiation into dopaminergic neurons using glial derived factors [24].

Species-specific cell types derived from NHP-iPSCs may open the door to test transformative developmental, translational, or evolutionary hypotheses in a field of inquiry currently hampered by the limited availability of research specimens. Because of their similarities to humans, NHP are uniquely relevant to many disease models. They also can be used to address specific questions that can be studied back and forth between in vitro cellular assays and in vivo experimentations, an investigational process that in most cases cannot be performed on humans because of safety and ethical issues. 

Well-characterized and validated NHP-iPSCs are currently unavailable. This contributes to variability and discrepancies between laboratories and hinders repeatability of key findings and progress of stem cell research and development. Animal cell lines and models have historically made significant contributions to our understanding of human diseases. However, disparities between results in preclinical animal studies and clinical trials have been identified, including failure to acknowledge the limitations of animal species and disease models [25]. The possibility to generate specialized organ-specific differentiated cells from iPSCs has galvanized regenerative medicine. The naïve pluripotency state of the iPSCs is critical to their developmental potential. In addition to clonal variability, iPSCs may be initially primed or drift in culture and become biased toward a specific lineage and unable to fulfill the pluripotency criteria [26,27]. When iPSCs become primed, their potency to contribute to embryonic and extra-embryonic chimeric tissue after injection into a blastocyst becomes limited [28]. In fact, specific culture conditions have been developed to maintain the pluripotent stem cells (PSCs) in an extended PSC stage (EPSC) with widespread chimeric contribution to embryonic and extraembryonic lineages in vivo [28]. The derivation and tendency of the marmoset pluripotent stem cells to differentiate into neural lineages has been reported [29,30,31,32,33,34,35,36,37] using variable techniques for the neuralization based on the initial protocol developed for human embryonic stem cells [38,39,40,41,42,43]. In this study, we demonstrated that using the NN1 medium supplemented with growth factors, we were able to isolate NSCs that are responsive to mitogenic growth factor. These data suggests that, like human pluripotent stem cells [43], the marmoset iPSCs harbor growth factor-responsive NSCs that grow as neurospheres. Interestingly, the marmoset NSCs responded to the glial-derived instructive cues to express the dopaminergic lineage, which was similar to brain-derived and hESC-derived NSCs [24,44]. Furthermore, recent studies have reported that neural cells derived from marmoset iPSCs have the potential to differentiate into the floor plate and generate dopaminergic neurons [29], similarly to the hESCs [45]. Together, these data suggest that the marmoset iPSCs may respond to extrinsic cues in a similar fashion to ESC; however, further optimization and time-course analysis would be necessary to explore the full potential of the NHP-iPSCs.

Previous studies have suggested that results from teratomas may be variable and provide limited quantitative information to make decisions on the quality of the chosen lines [18,19,20]. Genomic approaches [21,22] have been used as a quantitative and quick way to assess the quality and functional pluripotency of stem cell lines. Two genomic assays have been reported. The PluriTest [21] measures the molecular signature of pluripotency and uses this to classify pluripotent samples with great sensitivity and specificity. The second is the Scorecard [22,23], which evaluates both the molecular signature of pluripotency and the expression signatures that indicate functional pluripotency, defined as differentiation into each of the three germ layers; namely, ectoderm (EC), mesoderm (ME), and endoderm (ED). The Scorecard developed for pluripotent stem cells [22,23] is a gene expression signature assay based on the expression profiling of lineage-specific genes in differentiating embryoid body (EB) and on comparison to reference transcriptome maps generated using several ESC and iPSC lines. Its advantage is that it may be used to validate iPSCs for both pluripotency and for differentiation potential into EC, ME, and ED. Both of the iPSC lines we derived passed the scorecard test, which demonstrated not only the capacity of these iPSCs to generate the three germ layers, but also quantifies the tendency of iPSCs differentiation bias toward ectoderm, mesoderm, and endoderm. Furthermore, the molecular signature generated by the scorecard served as an identity marker of the cell line in addition to karyotyping. Other methods of authentication include DNA fingerprinting and PCR based short tandem repeats (STRs) analysis. The STRs may be used to document the authenticity and traceability of the cell line by confirming that it matches the genetic identity of the starting material. We have also validated the potential of these iPSCs to generate NSCs. The derived NSCs grew in suspension and displayed morphologies of spheres or organoids similar in shape to the fetal brain-derived NSCs [24], suggesting their multipotency to differentiate into neural tissue. Further in vivo assay in animal models is the next step to validate the developmental potential and the functional integration into host tissue.

## 4. Materials & Methods

### 4.1. Generation of Marmoset iPS Cells from Skin Biopsy

The marmosets (*Callithrix jacchus*) were from the Southwest Nonhuman Primate Research Center (SNPRC) colony. The SNPRC is registered and authorized by local and regional governmental authorities and accredited by the Association for Assessment and Accreditation of Laboratory Animal Care International (AAALAC). All procedures involving animals were performed in compliance with the guide for care and use of laboratory animals regulated by the Office of Laboratory Animal Welfare (OLAW) at the National Institutes of Health. The experimental procedures were approved by the Texas Biomedical Research Institute Institutional Animal Care and Use Committee (IACUC 1461 CJ-1, 16 December 2014). From selected marmosets, four to eight years old with good health confirmed, skin biopsies (4 to 8 mm), were taken from the back of animals by the veterinary staff according to standard operating procedures (SOP). The marmoset iPS cells were derived and characterized as outlined in Figure 1:

1. The biopsies were transported to our cell culture facility, minced into small pieces (1 mm), and cultured in Dulbecco’s modified Eagle’s medium (DMEM) supplemented with 20% of fetal bovine serum (FBS, Hyclone, ThermoFisher Scientific, Waltham, MA, USA) and 1% of penicillin/streptomycin (Gibco, ThermoFisher Scientific, Waltham, MA, USA).
2. The fibroblast cultures were usually confluent after 2–3 weeks, passaged once, and tested for the presence of mycoplasma using Hoechst 33258 DNA staining and the mycoplasma PCR detection Kit (Sigma-Aldrich, St. Louis, MO, USA).
3. Quality control checks also confirmed freedom from advantageous agents and viability superior to 80%. The selected fibroblasts were expanded for two to five passages to freeze a stock and to transfect with the reprogramming factors.
4. Nucleofection of the reprogramming factors: the fibroblasts passage numbers 3 to 6 were trypsinized and five hundred thousand fibroblasts were nucleofected with human episomal plasmids (pCXLE-hOCT3/4-shp53-F, pCXLE-hSK, pCXLE-hUL, and pCXWB-EBNA1; Addgene plasmid #27077, 27078, 27080, and 37624) using the Amaxa Nucleofector device and neonatal human dermal fibroblasts (NHDF) Nucleofector kit Program U-023 (Lonza, Walkersille, MD, USA).
5. Nucleofected fibroblasts were transferred onto an irradiated mouse embryonic fibroblasts (MEF) feeder and incubated for three days in DMEM/F12, 20% KnockOut Serum Replacement, 2 mM l-glutamine, MEM Non-Essential Amino Acids Solution, β-Mercaptoethanol (all from Gibco, ThermoFisher Scientific), and 10 ng/mL basic fibroblast growth factor (bFGF, Stemgent, Beltsville, MD, USA). The cultures were maintained for five weeks until the colonies emerged.
6. When human embryonic stem cell like colonies appeared, they were isolated, dissociated by pipetting, and transferred into a single well on an irradiated MEF feeder. The H9 hESCs were also cultured on feeder layer as we previously reported [43].
7. Validated and authenticated iPSC lines were scaled up to generate 100 vials of 5 to 10 million cell/vial cryopreserved as a seed master cell bank (MCB) for each iPSC line. The iPSCs were cryopreserved in their growth media supplemented with 40% serum and 10% dimethylsulfoxide (DMSO) as cryprotectant.
8. One vial of the iPSCs taken from the MCB is used to create a working cell bank.

### 4.2. Complementary DNA Synthesis, Q-PCR Analysis, and TaqMan Q-PCR Analysis

Undifferentiated CJ01 and CJ02 iPSC lines and H9 hESCs were cultured confluent on six-well plates and collected using cell scrapper. RNAs were isolated using RNeasy-Plus Mini-kit (QIAGEN, Germantown, MD, USA) according to the manufacture’s protocol. C-DNAs were synthesized from 1 μg of RNA using SuperScript IV First-Strand Synthesis system (Invitrogen, Carlsbad, CA, USA), according to the manufacturer’s protocol. Q-PCR analysis was conducted using SYBR Green PCR Master Mix (Applied Biosystems, Foster City, CA, USA). Q-PCR primers for detection of reprogramming factors were previously reported [17]. Coding region-specific primers are *OCT3/4:* CCC CAG GGC CCC ATT TTG GTA CC/ACC TCA GTT TGA ATG CAT GGG AGA GC; *SOX2:* TTC ACA TGT CCC AGC ACT ACC AGA/TCA CAT GTG TGA GAG GGG CAG TGT GC; KLF4: ACC CAT CCT TCC TGC CCG ATC AGA/TTG GTA ATG GAG CGG CGG GAC TTG; *L-MYC:* GCG AAC CCA AGA CCC AGG CCT GCT CC/CAG GGG GTC TGC TCG CAC CGT GAT G and *LIN28:* AGC CAT ATG GTA GCC TCA TGT CCG C/TCA ATT CTG TGC CTC CGG GAG CAG GGT AGG.

Primers specific for the coding region of the pluripotent genes were used to check for their expression by the iPSCs generated and to confirm whether the sequences matched the marmoset gene sequences. Fibroblasts were used as negative control for the expression of pluripotency genes. For the plasmid vector-specific primers; *OCT3/4:* CAT TCA AAC TGA GGT AAG GG/TAG CGT AAA AGG AGC AAC ATA G, *SOX2:* TTC ACA TGT CCC AGC ACT ACC AGA/TTT GTT TGA CAG GAG CGA CAA T, *KLF4:* CCA CCT CGC CTT ACA CAT GAA GA/TAG CGT AAA AGG AGC AAC ATA G, *L-MYC:* GGC TGA GAA GAG GAT GGC TAC/TTT GTT TGA CAG GAG CGA CAA T and *LIN28:* AGC CAT ATG GTA GCC TCA TGT CCG C/TAG CGT AAA AGG AGC AAC ATA G.

The Q-RT-PCR experiment was conducted in technical duplicates and the fold of increase was expressed as ratio of the Ct of the coding region to the Ct of H9 cells and those for the plasmid-specific to the Ct of Day-3 culture as positive control.

The Q-PCR for the NSCs and dopaminergic cell markers was performed using the following TaqMan gene expression assays (ThermoFisher Scientific): *NES* (Hs00707120_s1), *MBP* (Hs00921945_m1), *SOX1* (Hs01057642_s1), *SOX2* (Hs01053049_s1) and *PAX6* (Hs01088112_m1); *TH* (H200165941_m1), *LMX1A* (Hs00892663_m1), *DDC* (Hs01105042_m1), *EN1* (Hs00154977_m1), *NR4A2* (Hs00428691_m1) and *PITX3* (Hs00374504_m1).

### 4.3. Embryoid Body (EB) Formation and Scorecard Analysis

The Scorecard analysis was performed as outlined in Figure 4:

1. CJ01 and CJ02 iPSC lines and the H9 hESC line were cultured confluent in six-well plates and the colonies were collected using cell scrapper and cultured in EB media (DMEM/F12, 20% KnockOut Serum Replacement, 1 mM Non-Essential Amino Acids Solution, 55 μM β-Mercaptoethanol).
2. The cell suspensions were plated on non-adhesive six-well plates for seven days. Media was changed every two days.
3. On the seventh day, the EB’s suspension was collected and RNA was extracted using RNeasy-Plus Mini-kit (QIAGEN).
4. Synthesize of the cDNAs was performed using the SuperScript IV First-Strand Synthesis system (Invitrogen).
5. The cDNAs were used in the TaqMan hPSC Scorecard Panel (ThermoFisher Scientific) with TaqMan Fast Advanced Master Mix (ThermoFisher Scientific) as per the manufacturer’s protocol.
6. Data showing the relative levels of self-renewal genes, mesodermal genes, ectoderm, and endodermal genes are analyzed using the cloud-based software provided with the Scorecard.
7. Analyzed data shown a pass or fail results of the pluripotency test, indicating whether the cell line is pluripotent or biased to any of the germ layers.

### 4.4. Generation of NSCs and Differentiation into Dopaminergic Neurons:

CJ01 and CJ02 iPSCs were cultured on a feeder layer of mouse embryonic fibroblasts in DMEM-F12 (ThermoFisher Scientific), with 20% Knockout serum replacement (ThermoFisher Scientific), 10 ng/mL basic fibroblast growth factor (bFGF, Stemgent), 1% non-essential amino acids (ThermoFisher Scientific), and 0.5% l-glutamine (containing 0.14% 2-mercaptoethanol). The media was changed daily and cells were passaged once every five days. For the generation of neurospheres, confluent cultures were detached non-enzymatically using a cell lifter (ThermFisher Scientific), rinsed twice with PBS, and cultured in NN1 medium (NeoNeuron, San Antonio, TX, USA) containing 20 ng/mL bFGF and 20-ng/mL epidermal growth factor (EGF, EMD Millipore, Burlington, MA, USA). The NSCs were dissociated into single cells with accutase (ThermoFisher Scientific) and expanded on a weekly basis.

The dopaminergic differentiation was induced by culturing the NSCs for one week in NN1 medium containing EGF, bFGF, and retinoic acid (2 μM). On the day of differentiation (day 0), the neurospheres were collected by centrifugation (1000 RPM for 5 min) and rinsed twice with NN1 medium. The neurospheres were resuspended in media containing 75% (*v*/*v*) DIF-1 (dopamine inducing factor-1, NeoNeuron), 25% (*v*/*v*) NN1 medium 10 ng/mL bFGF, 200 μM ascorbic acid (Sigma), and 1% fetal bovine serum (FBS, Hyclone) and plated on poly-l-ornithine (0.16 μg/mL, Sigma) coated glass cover slips. The cells were replenished with fresh media every 24 h. On day 4 of differentiation, the cells were switched to media containing 25% of Dopamine Inducing Factor 1 (DIF1, NeoNeuron, San Antonio, TX, USA), 75% NN1 medium, 10 ng/mL bFGF, 200 μM ascorbic acid, and 1% FBS. For control wells, NN1 medium with + 1% FBS was used and media was changed every 24 h. On day 7 of differentiation, the cells were fixed with 4% paraformaldehyde and processed for immunocytochemistry.

### 4.5. Immunocytochemistry

Cultures were fixed with 4% paraformaldehyde for 15 min and rinsed in phosphate buffered saline (PBS) for 3 × 5 min and then incubated for two hours with the appropriate primary antibodies for multiple labeling. Secondary antibodies raised in the appropriate hosts and conjugated to FITC, RITC, AMCA, CY3, or CY5 fluorescent or chromogenes (Jackson ImmunoResearch, West Grove, PA, USA) were used. The total plated cells were counterstained with the nuclear marker 4′,6-diamidine-2-phenylindole dihydrochloride (DAPI) (ThermoFisher Scientific). Positive and negative controls were included in each run and in our validated assays. The H9 hESCs were used as positive control and the negative controls included the omission of primary antibodies, and as a second control, we included fixed cells without any antibodies to identify any potential auto-fluorescence. Immunostained sections were coverslipped using fluorsave (Calbiochem, Burlington, MA, USA) as the mounting medium. The following antibodies were used: Anti-TH (1:100, Pel-Freez, Rogers, AR, USA, P40101-150) or β Tubulin (1:500, Sigma, T8660), Anti-OCT3/4 (1:200, Millipore, Burlington, MA, USA), Anti-NANOG clone7F7.1 (1:500, Millipore), Anti-TRA-1-60 (1:100, Stemgent), and Anti-SSEA4 (1:100, BD Pharmingen, San Jose, CA, USA). Immunostained cells were coverslipped using fluorsave (Calbiochem) as the mounting medium and analyzed using the LSM-800 ZEISS laser scanning confocal microscope (Thornwood, NY, USA). Dopamine neurons quantitative analysis: images of 6–8 random fields from day 7 immunofluorescence labelled cultures were taken using the Zeiss LSM 800 confocal microscope. The total number of DAPI positive, TUJ1 positive, and TH positive cells were counted manually using the Image-J (National Institutes of Health (NIH, https://imagej.nih.gov/ij/) software. The cell counts were exported to excel and plotted using Graph Pad Prism statistical software (La Jolla, CA, USA). The total number of neurons (TUJ1 positive) and dopamine neurons (TUJ1 and TH double positive) was expressed as a percentage of DAPI positive cells.

### 4.6. Alkaline Phosphatase (AP) Staining

The culture medium was aspirated and colonies washed with PBS supplemented with 0.05% Tween 20 (PBST, Sigma). The iPSC colonies were fixed for 5 min at room temperature and washed with PBST. The cells were then incubated with the AP substrate solution in the dark for 5 to 15 min at room temperature while monitoring the color change. When the color turned red/purple stain, the reaction was stopped by aspirating the AP substrate solution and washing the wells were washed twice with PBS. The cells were covered with PBS and stored at 4 °C.

### 4.7. Karyotyping

The cells were incubated at 37 °C in a 5% CO_2_ incubator for 1 h in culture medium supplemented with Colcemid (ThermoFisher Scientific) at concentration of 0.1 µg/mL. The cells were detached from plate using accutase and gently dissociated and incubated in 0.075 M KCl (Sigma) for 15 to 20 min. The cells were fixed and washed three times before homogenization. From the homogenate, 20–30 μL of cell suspension were plated onto a clean dry slide and left to dry. The slides were aged in a dry oven at 55 to 60 °C overnight. The slides were then stained with Giemsa solution (Sigma) and coverslip with cytoseal (Richard-Allan Scientific, San Diego, CA, USA). A total of 20 cells per culture every 5 to 10 passages were screened for each cell line for microscopic aberrations of over 5 Mb chromosomes, including aneuploidy, inversions, duplications, deletions, and translocations.

### 4.8. Statistical Analysis

Statistical analysis was done with Graph Pad Prism statistical software. Significance in differences between two groups was performed by applying Student’s *t*-test where appropriate. For comparison of multiple groups one-way or two-way analysis of variance (ANOVA) with Newman–Keuls multiple comparison test or Bonferroni post-hoc analysis was performed to identify the significant differences. A *p*-value of less than 0.05 was considered to be statistically significant.

## 5. Conclusions

In conclusion, we describe defined standards for establishing iPSC lines from NHP. Such standards are critical in scientific research to generate reliable high quality iPSCs. A standardized resource of NHP-iPSCs will enhance rigor and reproducibility of study results and play a unique role in cell biology, genetics, and regenerative medicine by providing an unlimited source of differentiated derivatives that can be used to address important questions in comparative biology and medicine. Additionally, when used as a standardized source of cells for regenerative medicine, NHP-iPSCs serve an invaluable role in translational allogeneic and autologous experimental cell therapy. As NHPs are the closest to humans in the complexity of the immune system, this iPSC resource will enable future experiments investigating the interplay between grafts and host in functional recovery.
